# Influence of arteriovenous fistula on daily living behaviors involving the upper limbs in hemodialysis patients: a cross-sectional questionnaire study

**DOI:** 10.1186/s12882-018-1097-9

**Published:** 2018-10-22

**Authors:** Yuuta Hara, Kosuke Sonoda, Koji Hashimoto, Kazuaki Fuji, Yosuke Yamada, Yuji Kamijo

**Affiliations:** Department of Nephrology, Shinshu University School of Medicine, 3-1-1 Asahi, Matsumoto, Nagano, 390-8621 Japan

**Keywords:** Arteriovenous fistula, Dialysis, Dominant arm, Living behavior, Motor function

## Abstract

**Background:**

Arteriovenous fistulae can restrict daily living behaviors involving the upper limbs in hemodialysis patients, but no studies have investigated the detailed effects of an arteriovenous fistula on routine life activities. Accordingly, many medical caregivers are unable to explain the effects of an arteriovenous fistula on daily life, particularly during non-dialysis periods, because they cannot observe them directly.

**Methods:**

Thirty outpatients undergoing hemodialysis at 2 facilities scored the difficulty due to an arteriovenous fistula in performing 48 living behaviors during non-dialysis and 10 behaviors during dialysis into 5 grades in a comprehensive questionnaire survey. These behaviors were selected based on an open-answer pre-questionnaire administered to the 30 patients beforehand. The scores were also compared between dominant arm and non-dominant arm arteriovenous fistula groups.

**Results:**

During non-dialysis, the difficulty scores of behaviors restricted out of concern for arteriovenous fistula obstruction (wear a wristwatch, hang a bag on the arm, carry a baby or a dog in the arms, wear a short-sleeved shirt, etc.) increased. The difficulties of “wear a wristwatch” and “hang a bag on the arm” were significantly higher in the non-dominant arm arteriovenous fistula group (both *P* < 0.05). In contrast, scores related to motor function (write, eat or drink, scratch an itch, etc.) increased remarkably during dialysis because of connection of the arteriovenous fistula to the dialysis machine. The difficulties of “write” and “eat or drink” were significantly higher in the dominant arm arteriovenous fistula group (both *P* < 0.05).

**Conclusions:**

Several key daily living behaviors restricted by an arteriovenous fistula were identified in this questionnaire survey. These results will be useful for pre-operative explanation of arteriovenous fistula surgery and arm selection in end-stage renal disease patients.

**Electronic supplementary material:**

The online version of this article (10.1186/s12882-018-1097-9) contains supplementary material, which is available to authorized users.

## Background

The number of chronic kidney disease patients has been increasing due to lifestyle diseases and an aging population, with those reaching end-stage renal disease requiring the induction of hemodialysis. At that time, many patients require the creation of an arteriovenous fistula (AVF) on either forearm [1, 2].

Once an AVF is made, many patients experience difficulties in using their upper limbs [3] and suffer a decline in quality of life [4], firstly since they must constantly protect the AVF even during non-dialysis [1, 2], secondly because of decreased cosmesis due to vasodilation [5], thirdly as the range of upper-limb motion may become limited from the dilated blood vessel and modification of blood flow [6], and fourthly because moving the arm during dialysis becomes impaired by connection to the dialysis machine. The above issues represent a severe problem for many individuals, who understandably feel uneasy at pre-AVF consultation [7]. However, there are currently no studies investigating precisely how an AVF affects living behaviors. For that reason, many medical providers are unable to give detailed explanations on the influence of AVFs on lifestyle, particularly during non-dialysis, because they cannot observe them. This may fuel patient anxiety when deciding on AVF creation. Qin et al. described that professional strategies of internal fistulae could prolong service time, decrease complications, and increase quality of life [8]. We therefore devised a comprehensive questionnaire to identify which living behaviors were affected most by AVFs in hemodialysis patients. Moreover, we statistically investigated for differences in having the AVF in the dominant or non-dominant arm, a question often discussed at AVF consultations.

## Methods

### Study design

This was a cross-sectional questionnaire study.

### Study patients

Forty-two Japanese patients over the age of 20 years and undergoing outpatient maintenance hemodialysis at either of 2 dialysis clinics (Kanno Dialysis and Vascular Access Clinic or Jishukai Ueda Kidney Clinic) who fulfilled the eligibility criteria below were approached. The inclusion criteria were: 1) currently receiving hemodialysis via an AVF, 2) performance status (PS) [9] of 0 or 1, and 3) having received at least 1 month of regular dialysis. The exclusion criteria were: 1) currently receiving hemodialysis via a non-AVF site, such as an arteriovenous graft, subcutaneously fixed superficial artery, or permanent vascular catheter, 2) having functional AVFs on both arms, 3) having impaired upper limb function due to problems other than AVF, such as hemiplegia, carpal-tunnel syndrome, or traumatic injury, 4) questionnaire response was difficult owing to dementia or a psychiatric disorder, and 5) ambidexterity. Twelve patients who did not provide consent to participate were excluded. The remaining 30 subjects were enrolled for this questionnaire study.

The subjects were analyzed for age, dialysis duration, gender, PS, dominant arm, number of AVF creations, occupation, cause of kidney disease, anastomotic site, AVF vessel size category (0, obscure; 1, thinner than the digitus minimus; 2, thicker than the digitus minimus but thinner than the thumb; 3, thicker than the thumb), and AVF after-effects such as steal syndrome and/or sore finger syndrome. The above information was collected from medical records or direct interviews with the patient.

### Method for producing the questionnaire

The living behaviors evaluated in the questionnaire were selected to include activities indispensable in daily life that were affected by the presence of an AVF. To identify such behaviors, a preliminary, open-answer questionnaire (see Additional file 1) was administered to all 30 patients, asking: “Please list as many living behaviors outside of dialysis room as possible that are restricted by your AVF” and “Please list as many living behaviors as possible that are restricted due to your AVF being connected to the dialysis machine during treatment”. Based on these results, 19 living behaviors during non-dialysis were identified: wear a wristwatch, carry a heavy object (over 5 kg), wear a short-sleeved shirt, drive a car, hang a bag on the arm, wear wrist-constricting clothes, bend the arm for an extended time, sleep in an unrestricted position, hold a handle strongly, enter a hot spring or public bath, carry a baby or dog in the arms, receive an arm massage, *care not to rub the arm strongly, *care not to hit the arm, *concern the AVF is obstructed due to dehydration, *care to avoid insect bites on the arm, *puncture site itchiness, *listlessness in the shoulder after dialysis, and *care to protect the arm from becoming cold. Five living behaviors during dialysis (eat or drink, operate a TV remote controller, sleep in an unrestricted position, read a book, and write) were listed as well.

To ensure an exhaustive list of living behaviors indispensable in daily life, 26 behaviors were selected according to the Disability of the Arm, Shoulder, and Hand (DASH) score [10], a general upper limb function evaluation tool used in the orthopedic field. As behaviors during non-dialysis, the following items were added: open a tight or new jar, write, turn a key, prepare a meal, push open a heavy door, place an object on a shelf above your head, do heavy household chores, garden or do yard work, make the bed, carry a shopping bag or briefcase, change a lightbulb overhead, wash or blow dry your hair, wash your back, put on a pullover sweater, use a knife to cut food, do recreational activities which require little effort (ex., playing cards, knitting, playing Japanese board games), do recreational activities in which you take some force or impact through your arm, shoulder, or hand (ex., golfing, playing tennis, playing catch ball, using a hammer), do recreational activities in which you move your arm freely (ex., throwing a flying disc, playing badminton), manage transportation needs, and engage in sexual activities. We also asked about *pain in the arm, shoulder, or hand at rest apparently caused by the AVF, *difficulty sleeping due to pain in the arm, shoulder, or hand apparently caused by the AVF, *pain in the arm, shoulder, or hand while performing any specific activity apparently caused by the AVF, *weakness in the arm, shoulder, or hand apparently caused by the AVF, *stiffness in the arm, shoulder, or hand apparently caused by the AVF, and *feel less capable, confident, or useful because of the AVF.

Five doctors in the dialysis field added 3 behaviors during non-dialysis (hold a pot, perform a blood pressure check, and do self-hemostasis of the AVF) and 5 behaviors during dialysis (operate a mobile phone or smart phone, communicate with staff or other patients, remove something from your bag, take medicine, and scratch an itch). Ultimately, these 48 items during non-dialysis and 10 items during dialysis were included in the final questionnaire (see Additional file 2).

The 48 items during non-dialysis were subdivided into 35 items related to activities and 13 items related to symptoms and feelings (indicated above by an asterisk). For the items related to activities, the questionnaire stated “Please rate how much the AVF disturbed the following activities in the past week. If you did not have the opportunity to perform an activity in the past week, please give your best estimate on which response would be the most accurate. It does not matter which hand or arm you used to perform the activity; please answer based on your ability regardless of how well you performed the task. For example, if you wrote with the right hand before having your AVF but are currently writing with the left hand because of the AVF, answer on the ability of writing with the left hand.” The patients answered the questionnaire using 5 grades: 1) no difference with the AVF, 2) mild difficulty due to the AVF, 3) moderate difficulty due to the AVF, 4) severe difficulty due to the AVF, and 5) not possible due to the AVF. For the items related to symptoms and feelings, the questionnaire stated: “Please rate the severity of the following symptoms and feelings in the past week.” The patients answered the questionnaire using 5 grades: 1) none, 2) mild, 3) moderate, 4) severe, and 5) extreme so I could do nothing.

All 10 items during dialysis were related to activities. The questionnaire stated: “Please rate how much the AVF disturbed the following activities during dialysis in the past week. In this part, AVF means AVF connected to the dialysis machine. If you did not have the opportunity to perform an activity in the past week, please give your best estimate on which response would be the most accurate. It does not matter which hand or arm you used to perform the activity; please answer based on your ability regardless of how well you performed the task.” The grading was identical to that for the non-dialysis activities.

### Method for completing the questionnaires

A written questionnaire was given to all of the subjects, who completed it by themselves during or after dialysis. Since the outcomes contained subjective evaluation, there was a possibility of result bias, particularly when a researcher could easily identify a specific individual. Therefore, the surveys were recorded by means of an anonymous identification number for each respondent.

### Statistical analysis

For patient characteristics, qualitative data are expressed as the number (percentage) and quantitative data are presented as the median (range). Regarding questionnaire scores, the average of all scores was calculated for each item. Missing data were excluded from the analysis. Response rates (RR) were calculated and shown. Additional comparison between the dominant-arm AVF group (DA-group) and non-dominant arm AVF group (nDA-group) were performed using the chi-square test for qualitative data and the Mann-Whitney U test for quantitative data. Statistical significance was defined as *P* < 0.05 as calculated by IBM SPSS statistics version 20 software (IBM Co., New York, USA).

## Results

Table 1 summarizes the patient characteristics. There were 30 patients in total (15 each in the DA-group and nDA-group) with a variety of occupations, age range of 40 to 83 years, and dialysis duration range of 1 to 403 months. No patient had steal syndrome or sore finger syndrome. There were no significant differences between the test groups.

**Table 1:** Characteristics of the study patients

	All patients *N* = 30	Dominant-arm AVF group *N* = 15	Non-dominant arm AVF group *N* = 15	*P*-value
Age (years)	63.5 (48–83)	63.0 (48–81)	64.0 (47–83)	0.65
Dialysis duration (months)	48.5 (1–403)	31.0 (1–331)	51.0 (5–403)	0.33
Gender (male)	22 (73%)	11 (73%)	11 (73%)	1.00
Performance status (0: 1)	26: 4 (87%: 13%)	13: 2 (87%: 13%)	13: 2 (87%: 13%)	1.00
Dominant arm (right)	29 (97%)	15 (100%)	14 (93%)	1.00
Number of AVF creations	1 (1–3)	1 (1–3)	1 (1–2)	0.49
Occupation				
Blue-collar worker	7 (23%)	4 (27%)	3 (20%)	1.00
White-collar worker	5 (17%)	2 (13%)	3 (20%)	1.00
Unemployed	18 (60%)	9 (60%)	9 (60%)	1.00
Cause of kidney disease				
Diabetes mellitus	13 (43%)	7 (47%)	6 (40%)	1.00
Chronic glomerular nephritis	13 (43%)	7 (47%)	6 (40%)	1.00
Other	4 (14%)	1 (6%)	3 (20%)	0.60
Anastomotic site				
Anatomical snuffbox	5 (17%)	2 (13%)	3 (20%)	1.00
Distal forearm	21 (70%)	11 (74%)	10 (67%)	1.00
Middle forearm	4 (13%)	2 (13%)	2 (13%)	1.00
Proximal forearm	0 (0%)	0 (0%)	0 (0%)	–
Vessel size category of AVF^a^				
Distal forearm	1 (0–3)	1 (0–2)	1 (0–3)	0.17
Middle forearm	1 (0–3)	0 (0–3)	1 (0–3)	0.15
Proximal forearm	1 (0–3)	1 (0–3)	1 (0–2)	0.33
After-effect of AVF creation				
Steal syndrome	0 (0%)	0 (0%)	0 (0%)	–
Sore finger syndrome	0 (0%)	0 (0%)	0 (0%)	–
Other	0 (0%)	0 (0%)	0 (0%)	–

*AVF* arteriovenous fistula. Data are presented as the number (percentage) or median (range).

^a^: 0) obscure, 1) thinner than the digitus minimus, 2) thicker than the digitus minimus but thinner than the thumb, 3) thicker than the thumb

**Fig. 1:**
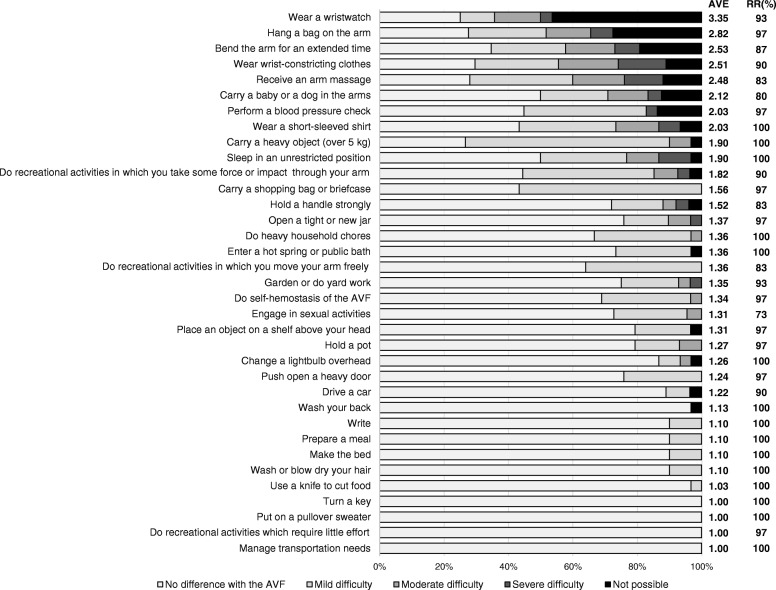
Results of graded evaluation of activities during non-dialysis. AVF: Arteriovenous Fistula, AVE: Average, RR: Response Rate

**Fig. 2:**
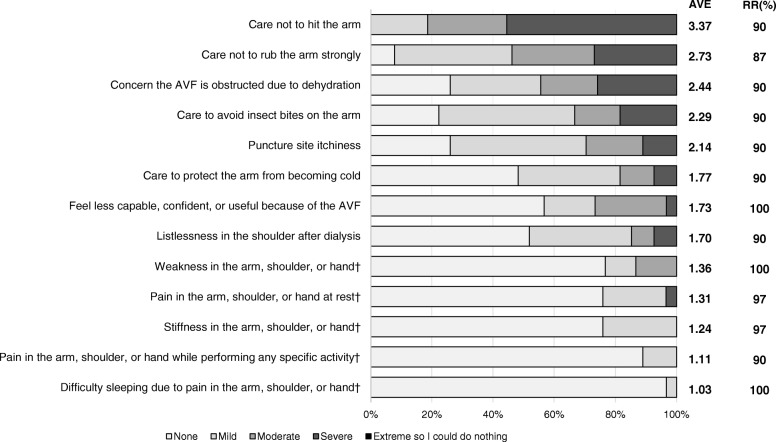
Results of graded evaluation of symptoms and feelings during non-dialysis. †, abbreviated by removing the term “apparently caused by the AVF”. AVF: Arteriovenous Fistula, AVE: Average, RR: Response Rate

**Fig. 3:**
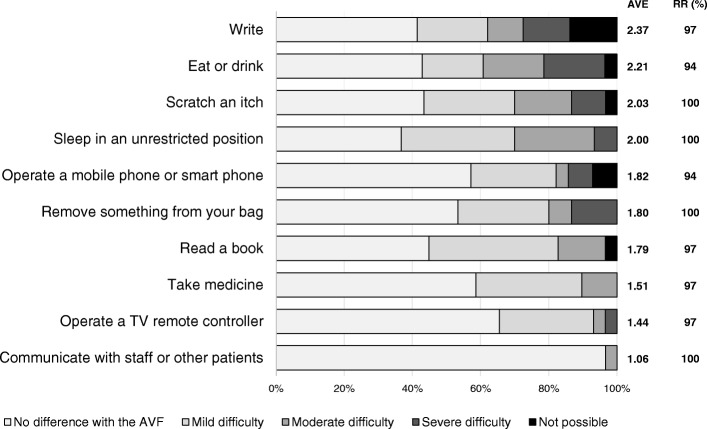
Results of graded evaluation of activities during dialysis. AVF: Arteriovenous Fistula, AVE: Average, RR: Response Rate

The overall results for living behaviors during non-dialysis and dialysis ranked according to average score value are presented in Figs. 1, 2 and 3. In items related to activity during non-dialysis (median RR: 97% [range: 73 to 100%]) (Fig. 1), the difficulty scores of items that could compress the AVF, such as “wear a wristwatch”, “hang a bag on the arm”, “bend the arm for an extended time”, “wear wrist-constricting clothes”, “receive an arm massage”, “carry a baby or a dog in the arms”, “perform a blood pressure check” and “wear a short-sleeved shirt” were relatively higher. Lower scores were seen for items that did not risk compression of the AVF, such as “write”, “prepare a meal”, “make the bed”, “wash or blow dry your hair”, “use a knife to cut food”, “turn a key”, “put on a pullover sweater”, “use a knife to cut food”, “do recreational activities which require little effort”, and “manage transportation needs”. Regarding items related to symptoms and feelings during non-dialysis (median RR: 90% [range: 87 to 100%]) (Fig. 2), the scores of those related to AVF protection, such as “care not to hit the arm”, “care not to rub the arm strongly”, and “concern the AVF is obstructed due to dehydration”, were highest. Scores were lowest for items unrelated to protecting the AVF, such as “listlessness in the shoulder after dialysis”, “difficulty sleeping because of pain in the arm, shoulder, or hand apparently caused by the AVF”, and “pain in the arm, shoulder, or hand apparently caused by the AVF while performing any specific activity”. Among the items related to behaviors during dialysis (median RR: 97% [range: 94 to 100%]) (Fig. 3), the scores for “write”, “eat or drink”, and “scratch an itch” were highest, while that for “communicate with staff or patients” was lowest.

**Fig. 4:**
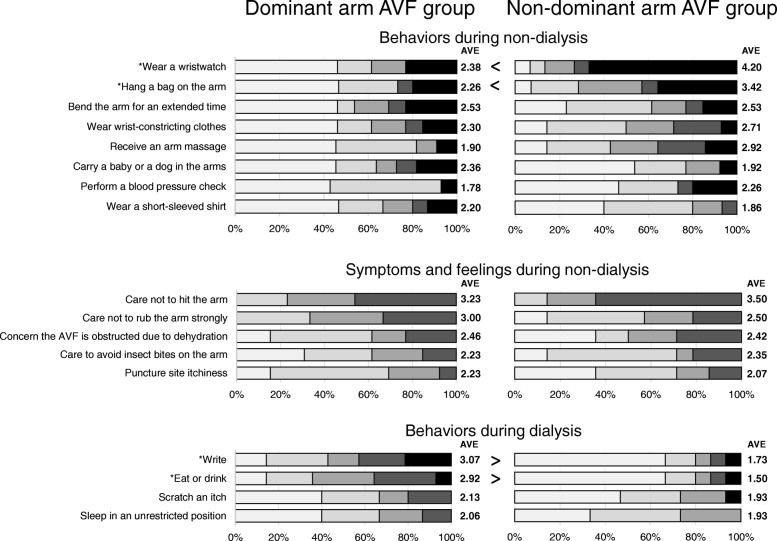
Comparison of scores between the dominant arm AVF group and non-dominant arm AVF group by the Mann-Whitney U test (*N* = 30). Selected items had an overall average score of 2 or more. AVF: Arteriovenous Fistula, AVE: Average. *, *P* < 0.05

The items with an average score of 2 (mild difficulty due to the AVF/mild) or more were next compared between the DA-group and nDA-group (Fig. 4). Concerning the items related to activity during non-dialysis, the scores for “wear a wristwatch” and “hang a bag on the arm” were significantly higher in the nDA-group (both *P* < 0.05). No significant differences were noted between the groups for symptoms and feelings during non-dialysis. Among the items related to activity during dialysis, the scores for “write” and “eat or drink” were significantly higher in the DA-group (both *P* < 0.05).

## Discussion

There have been no studies directly assessing the influence of AVFs on daily living behaviors involving the arms, which can be a serious problem for some hemodialysis patients. The present questionnaire survey revealed the most frequent difficulties among dialysis (activity-related) and non-dialysis (AVF compression avoidance-related) behaviors and clarified the possible differences in having an AVF in the dominant or non-dominant arm. Since AVF is the most often used type of vascular access [11, 12], this information will be very helpful for caregivers to better advise end-stage renal disease patients before and after the AVF procedure.

**Fig. 5:**
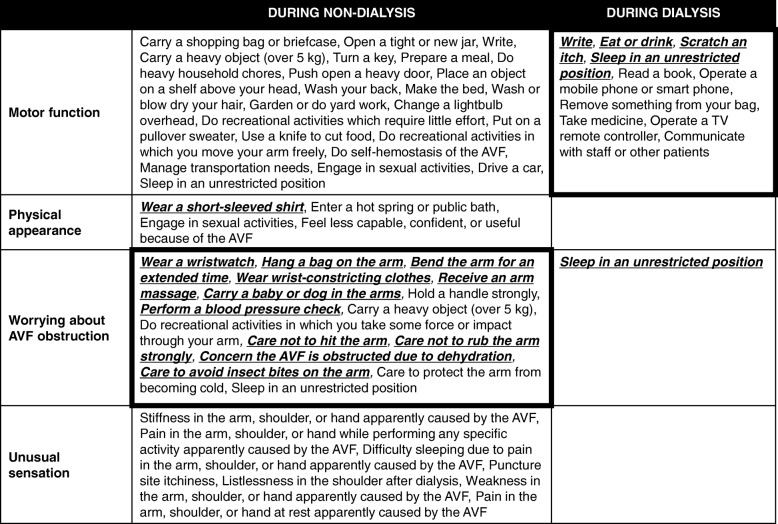
Questionnaire items classified into 4 categories: 1) those that do not compress the arm but become restricted when motor function of the arm declines (Motor function), 2) those related to physical appearance (Physical appearance), 3) those that can compress and/or damage the arm, causing restriction out of worry for AVF obstruction (Worrying about AVF obstruction), and 4) those related to unusual sensation (Unusual sensation). Bold, underlined, and italicized items had an overall average score of 2 or more. AVF: Arteriovenous Fistula

Based on our findings, it appeared possible to classify all tested items into 4 categories: 1) those that do not compress the arm but become restricted when motor function of the arm declines, 2) those related to physical appearance, 3) those that can compress and/or damage the arm, causing restriction out of worry for AVF obstruction, and 4) those related to unusual sensation (Fig. 5). In this context, the presence of an AVF had little influence on motor function itself during non-dialysis, with many living behaviors instead being restricted due to care for protecting the AVF. Indeed, protection of the AVF is essential for dialysis patients. Compressing the AVF vessel can cause obstruction [1] and wounds on the operated arm may lead to critical bleeding [13] .Additionally, since infection of the AVF site can sometimes be severe, patients need to keep the arm clean [14, 15]. Medical staff accordingly instruct patients to protect the AVF [1, 2], which seems to have the greatest influence on daily life. On the other hand, with little impact on the muscles and nerves, the AVF did not remarkably affect motor function among the respondents.

In the clinical context, patients scheduled to receive an AVF surgery will be better able to visualize post-operative life using the results of this study, which may reduce anxiety prior to surgery. Also, for individuals nervous about AVF protection, advocating long sleeves that do not constrict the AVF arm and/or an arm cover during non-dialysis periods may provide comfort. In contrast, motor function is more predominantly limited during dialysis because the arm is connected to the dialysis machine. Medical staff should therefore arrange the dialysis room environment such that patients can be entertained and perform behaviors not requiring specific work with their AVF arm during dialysis.

This is a novel study that highlights the influence of AVFs on the daily living behaviors of dialysis patients during non-dialysis, which is normally difficult for medical providers to observe in detail. In addition to increased scores for conventional items said to be avoided during AVF education, such as “hang a bag on the arm”, “wear a wristwatch”, “bend the arm for an extended time”, and “wear wrist-constricted clothes” [1], scores for many items important for communication and physical contact with family and friends, such as “carry a baby or a dog in the arms”, “receive an arm massage”, “wear a short-sleeved shirt”, and “do recreational activities in which you take some force or impact through your arm” were higher as well. Moreover, in items regarding symptoms and feelings during non-dialysis, nearly half of the cohort scored “feel less capable, confident, or useful because of the AVF” as 2 or more. The prevalence of depression is high in dialysis patients [16, 17], and worsened psychological status and quality of life have been associated with mortality [16–18] and the development of itchiness [19]. The presence of an AVF may make dialysis patients reluctant to communicate with others and represents a contributing factor to diminished psychological status, lower quality of life, and higher mortality. Careful monitoring for mental health is therefore advised.

In comparisons between the DA-group and nDA-group, the DA-group tended to have more difficulty with activities during dialysis while the nDA-group appeared to have more trouble with activities during non-dialysis. The dominant arm is generally used in a major role for exercise and fine work [20] and the non-dominant arm often plays a supplementary role, such as to wrap or hang an object on the arm or to immobilize objects [21]. As shown in Fig. 5, the AVF was connected to the dialysis machine during dialysis, thereby strongly affecting motor function. In contrast, it was difficult to perform behaviors such as wrapping or hanging objects on the arm during non-dialysis because of concern for AVF blockage. These findings may be beneficial when consulting patients on which arm is more suitable for an AVF; if the dominant arm that is responsible for primary motor function is selected, the difficulty scores during dialysis will tend to rise, while if the non-dominant arm that is responsible for supplementary roles is chosen, the scores during non-dialysis will likely increase. It is generally considered that the AVF should be made in the non-dominant arm considering behaviors during dialysis [1]. However, patients who regularly wear a wristwatch or hang things on the arm in their occupation or hobbies may instead be recommended to have the AVF in the dominant arm.

This study has several limitations. First, all participants were Japanese. Consideration of differences in race, religion, lifestyle, and physique will be needed when extrapolating these findings abroad. Second, patients who did not fulfill all eligibility criteria (especially poor PS or undergoing non-AVF dialysis) were excluded. Patients with poor PS have different lifestyles, and so our results may not have applied. Patients undergoing dialysis from a non-AVF site (especially those using catheters) may also not have been applicable to our results [22]. Moreover, the questionnaire was long and took considerable time to complete, causing some patients to decline participation. There was a possibility that only cooperative patients were selected for this study, which might have generated selection bias. Third, there were no patients with steal syndrome or sore finger syndrome resulting from their AVF in the study, which might limit the applicability of our results on patients with such after-effects of AVF creation. Fourth, the response rate was low for several question items (especially “engage in sexual activities”) that might have created information bias. Fifth, no controls were tested for comparisons with normal individuals. Lastly, particularly in the comparisons between the DA-group and nDA-group, statistical power may have been insufficient for some items due to small sample size. We are currently planning a larger, controlled study based on the items identified in this study.

## Conclusions

The presence of an AVF impairs some motor functions by connection to the dialysis machine during dialysis but generally does not affect motor behaviors during non-dialysis, at which time some activities are limited by worry about damage to the AVF. Patients having an AVF in the dominant arm tend to experience activity difficulties during dialysis, while those with an AVF in the non-dominant arm are more prone to restrictions during non-dialysis. The results of this study will help with patient explanation prior to AVF creation and more optimal selection of the AVF arm.

## Additional files

Additional file 1: The preliminary open-answer questionnaire. (DOCX 18 kb)

Additional file 2: The final questionnaire. (DOCX 33 kb)

## Abbreviations

AVF: Arteriovenous Fistula
DA-group: Dominant Arm group
DASH: Disability of the Arm, Shoulder, and Hand
nDA-group: non Dominant Arm group
PS: Performance Status
RR: Response Rates
