# Supplementary material for: Influence of arteriovenous fistula on daily living behaviors involving the upper limbs in hemodialysis patients: a cross-sectional questionnaire study
Source: BMC Nephrol. 2018 Oct 22;19:284. doi: 10.1186/s12882-018-1097-9 (PMC6198435; doi:10.1186/s12882-018-1097-9)
Supplement: Supplementary file 1 — The preliminary open-answer questionnaire. (DOCX 18 kb) [file 12882_2018_1097_MOESM1_ESM.docx]

**Additional File 1**

Survey on the Influence of Arteriovenous Fistula on Daily Living Behaviors

Department of Nephrology, Shinshu University School of Medicine

Identification number:　　　 .

We are investigating how much arteriovenous fistula (AVF) presence disturbs daily living behaviors, such as arm movement restrictions, appearance, desire to protect the AVF, etc.

Please answer the following questions by free description.

**1. Please list as many living behaviors outside of the dialysis room as possible that are restricted by your AVF. Please describe them specifically.**

**2. Please list as many living behaviors as possible that are restricted due to your AVF being connected to the dialysis machine during treatment. Please describe them specifically.**
