# Supplementary material for: Influence of arteriovenous fistula on daily living behaviors involving the upper limbs in hemodialysis patients: a cross-sectional questionnaire study
Source: BMC Nephrol. 2018 Oct 22;19:284. doi: 10.1186/s12882-018-1097-9 (PMC6198435; doi:10.1186/s12882-018-1097-9)
Supplement: Supplementary file 2 — The final questionnaire. (DOCX 33 kb) [file 12882_2018_1097_MOESM2_ESM.docx]

**Additional File 2**

Survey on the Influence of Arteriovenous Fistula on Daily Living Behaviors

Department of Nephrology, Shinshu University School of Medicine

Identification number :

We are investigating how much arteriovenous fistula (AVF) presence disturbs daily living behaviors, such as arm movement restrictions, appearance, desire to protect the AVF, etc. Please answer each question by circling your corresponding choice.

Which is your dominant arm? : Right / Left / Both

What is your current occupation? : Blue-collar worker / White-collar worker / Unemployed

**A) During non-dialysis**

**We will ask you about living behaviors during non-dialysis.**

**Please rate how much the AVF disturbed the following activities in the past week. If you did not have the opportunity to perform an activity in the past week, please give your best estimate on which response would be the most accurate. It does not matter which hand or arm you used to perform the activity; please answer based on your ability regardless of how well you performed the task. For example, if you wrote with the right hand before having your AVF but are currently writing with the left hand because of the AVF, answer on the ability of writing with the left hand**

**1.** **Open a tight or new jar**

|  | 1 : no difference with the AVF | 2 : mild difficulty due to the AVF | 3 : moderate difficulty due to the AVF | 4 : severe difficulty due to the AVF | 5 : not possible due to the AVF |
| --- | --- | --- | --- | --- | --- |

**2. Write**

|  | 1 : no difference with the AVF | 2 : mild difficulty due to the AVF | 3 : moderate difficulty due to the AVF | 4 : severe difficulty due to the AVF | 5 : not possible due to the AVF |
| --- | --- | --- | --- | --- | --- |

**3. Turn a key**

|  | 1 : no difference with the AVF | 2 : mild difficulty due to the AVF | 3 : moderate difficulty due to the AVF | 4 : severe difficulty due to the AVF | 5 : not possible due to the AVF |
| --- | --- | --- | --- | --- | --- |

**4. Prepare a meal**

|  | 1 : no difference with the AVF | 2 : mild difficulty due to the AVF | 3 : moderate difficulty due to the AVF | 4 : severe difficulty due to the AVF | 5 : not possible due to the AVF |
| --- | --- | --- | --- | --- | --- |

**5. Push open a heavy door**

|  | 1 : no difference with the AVF | 2 : mild difficulty due to the AVF | 3 : moderate difficulty due to the AVF | 4 : severe difficulty due to the AVF | 5 : not possible due to the AVF |
| --- | --- | --- | --- | --- | --- |

**6. Place an object on a shelf above your head**

|  | 1 : no difference with the AVF | 2 : mild difficulty due to the AVF | 3 : moderate difficulty due to the AVF | 4 : severe difficulty due to the AVF | 5 : not possible due to the AVF |
| --- | --- | --- | --- | --- | --- |

**7. Do heavy household chores (ex., wiping or floor cleaning, etc.)**

|  | 1 : no difference with the AVF | 2 : mild difficulty due to the AVF | 3 : moderate difficulty due to the AVF | 4 : severe difficulty due to the AVF | 5 : not possible due to the AVF |
| --- | --- | --- | --- | --- | --- |

**8. Garden or do yard work**

|  | 1 : no difference with the AVF | 2 : mild difficulty due to the AVF | 3 : moderate difficulty due to the AVF | 4 : severe difficulty due to the AVF | 5 : not possible due to the AVF |
| --- | --- | --- | --- | --- | --- |

**9. Make the bed**

|  | 1 : no difference with the AVF | 2 : mild difficulty due to the AVF | 3 : moderate difficulty due to the AVF | 4 : severe difficulty due to the AVF | 5 : not possible due to the AVF |
| --- | --- | --- | --- | --- | --- |

**10. Carry a shopping bag or briefcase**

|  | 1 : no difference with the AVF | 2 : mild difficulty due to the AVF | 3 : moderate difficulty due to the AVF | 4 : severe difficulty due to the AVF | 5 : not possible due to the AVF |
| --- | --- | --- | --- | --- | --- |

**11. Carry a heavy object (over 5 kg)**

|  | 1 : no difference with the AVF | 2 : mild difficulty due to the AVF | 3 : moderate difficulty due to the AVF | 4 : severe difficulty due to the AVF | 5 : not possible due to the AVF |
| --- | --- | --- | --- | --- | --- |

**12. Change a lightbulb overhead**

|  | 1 : no difference with the AVF | 2 : mild difficulty due to the AVF | 3 : moderate difficulty due to the AVF | 4 : severe difficulty due to the AVF | 5 : not possible due to the AVF |
| --- | --- | --- | --- | --- | --- |

**13. Wash or blow dry your hair**

|  | 1 : no difference with the AVF | 2 : mild difficulty due to the AVF | 3 : moderate difficulty due to the AVF | 4 : severe difficulty due to the AVF | 5 : not possible due to the AVF |
| --- | --- | --- | --- | --- | --- |

**14. Wash your back**

|  | 1 : no difference with the AVF | 2 : mild difficulty due to the AVF | 3 : moderate difficulty due to the AVF | 4 : severe difficulty due to the AVF | 5 : not possible due to the AVF |
| --- | --- | --- | --- | --- | --- |

**15. Put on a pullover sweater**

|  | 1 : no difference with the AVF | 2 : mild difficulty due to the AVF | 3 : moderate difficulty due to the AVF | 4 : severe difficulty due to the AVF | 5 : not possible due to the AVF |
| --- | --- | --- | --- | --- | --- |

**16. Use a knife to cut food**

|  | 1 : no difference with the AVF | 2 : mild difficulty due to the AVF | 3 : moderate difficulty due to the AVF | 4 : severe difficulty due to the AVF | 5 : not possible due to the AVF |
| --- | --- | --- | --- | --- | --- |

**17. Do recreational activities which require little effort**

**(ex., playing cards, knitting, playing Japanese board games)**

|  | 1 : no difference with the AVF | 2 : mild difficulty due to the AVF | 3 : moderate difficulty due to the AVF | 4 : severe difficulty due to the AVF | 5 : not possible due to the AVF |
| --- | --- | --- | --- | --- | --- |

**18. Do recreational activities in which you take some force or impact through your arm, shoulder, or hand**

**(ex., golfing, playing tennis, playing catch ball, using a hammer)**

|  | 1 : no difference with the AVF | 2 : mild difficulty due to the AVF | 3 : moderate difficulty due to the AVF | 4 : severe difficulty due to the AVF | 5 : not possible due to the AVF |
| --- | --- | --- | --- | --- | --- |

**19. Do recreational activities in which you move your arm freely**

**(ex., throwing a flying disc, playing badminton)**

|  | 1 : no difference with the AVF | 2 : mild difficulty due to the AVF | 3 : moderate difficulty due to the AVF | 4 : severe difficulty due to the AVF | 5 : not possible due to the AVF |
| --- | --- | --- | --- | --- | --- |

**20. Manage transportation needs**

|  | 1 : no difference with the AVF | 2 : mild difficulty due to the AVF | 3 : moderate difficulty due to the AVF | 4 : severe difficulty due to the AVF | 5 : not possible due to the AVF |
| --- | --- | --- | --- | --- | --- |

**21. Engage in sexual activities**

|  | 1 : no difference with the AVF | 2 : mild difficulty due to the AVF | 3 : moderate difficulty due to the AVF | 4 : severe difficulty due to the AVF | 5 : not possible due to the AVF |
| --- | --- | --- | --- | --- | --- |

**22. Wear a short-sleeved shirt**

|  | 1 : no difference with the AVF | 2 : mild difficulty due to the AVF | 3 : moderate difficulty due to the AVF | 4 : severe difficulty due to the AVF | 5 : not possible due to the AVF |
| --- | --- | --- | --- | --- | --- |

**23. Hang a bag on the arm**

|  | 1 : no difference with the AVF | 2 : mild difficulty due to the AVF | 3 : moderate difficulty due to the AVF | 4 : severe difficulty due to the AVF | 5 : not possible due to the AVF |
| --- | --- | --- | --- | --- | --- |

**24. Wear a wristwatch**

|  | 1 : no difference with the AVF | 2 : mild difficulty due to the AVF | 3 : moderate difficulty due to the AVF | 4 : severe difficulty due to the AVF | 5 : not possible due to the AVF |
| --- | --- | --- | --- | --- | --- |

**25. Enter a hot spring or public bath**

|  | 1 : no difference with the AVF | 2 : mild difficulty due to the AVF | 3 : moderate difficulty due to the AVF | 4 : severe difficulty due to the AVF | 5 : not possible due to the AVF |
| --- | --- | --- | --- | --- | --- |

**26. Drive a car**

|  | 1 : no difference with the AVF | 2 : mild difficulty due to the AVF | 3 : moderate difficulty due to the AVF | 4 : severe difficulty due to the AVF | 5 : not possible due to the AVF |
| --- | --- | --- | --- | --- | --- |

**27. Hold a pot**

|  | 1 : no difference with the AVF | 2 : mild difficulty due to the AVF | 3 : moderate difficulty due to the AVF | 4 : severe difficulty due to the AVF | 5 : not possible due to the AVF |
| --- | --- | --- | --- | --- | --- |

**28. Perform a blood pressure check**

|  | 1 : no difference with the AVF | 2 : mild difficulty due to the AVF | 3 : moderate difficulty due to the AVF | 4 : severe difficulty due to the AVF | 5 : not possible due to the AVF |
| --- | --- | --- | --- | --- | --- |

**29. Sleep in an unrestricted position**

|  | 1 : no difference with the AVF | 2 : mild difficulty due to the AVF | 3 : moderate difficulty due to the AVF | 4 : severe difficulty due to the AVF | 5 : not possible due to the AVF |
| --- | --- | --- | --- | --- | --- |

**30. Do self-hemostasis of the AVF**

|  | 1 : no difference with the AVF | 2 : mild difficulty due to the AVF | 3 : moderate difficulty due to the AVF | 4 : severe difficulty due to the AVF | 5 : not possible due to the AVF |
| --- | --- | --- | --- | --- | --- |

**31. Carry a baby or dog in the arms**

|  | 1 : no difference with the AVF | 2 : mild difficulty due to the AVF | 3 : moderate difficulty due to the AVF | 4 : severe difficulty due to the AVF | 5 : not possible due to the AVF |
| --- | --- | --- | --- | --- | --- |

**32. Wear wrist-constricting clothes**

|  | 1 : no difference with the AVF | 2 : mild difficulty due to the AVF | 3 : moderate difficulty due to the AVF | 4 : severe difficulty due to the AVF | 5 : not possible due to the AVF |
| --- | --- | --- | --- | --- | --- |

**33. Bend the arm for an extended time**

|  | 1 : no difference with the AVF | 2 : mild difficulty due to the AVF | 3 : moderate difficulty due to the AVF | 4 : severe difficulty due to the AVF | 5 : not possible due to the AVF |
| --- | --- | --- | --- | --- | --- |

**34. Hold a handle strongly**

|  | 1 : no difference with the AVF | 2 : mild difficulty due to the AVF | 3 : moderate difficulty due to the AVF | 4 : severe difficulty due to the AVF | 5 : not possible due to the AVF |
| --- | --- | --- | --- | --- | --- |

**35. Receive an arm massage**

|  | 1 : no difference with the AVF | 2 : mild difficulty due to the AVF | 3 : moderate difficulty due to the AVF | 4 : severe difficulty due to the AVF | 5 : not possible due to the AVF |
| --- | --- | --- | --- | --- | --- |

**Please rate the severity of the following symptoms and feelings in the past week.**

**36. Pain in the arm, shoulder, or hand at rest apparently caused by the AVF**

|  | 1 : none | 2 : mild | 3 : moderate | 4 : severe | 5 : extreme so I could do nothing |
| --- | --- | --- | --- | --- | --- |

**37. Pain in the arm, shoulder, or hand while performing any specific activity apparently caused by the AVF**

|  | 1 : none | 2 : mild | 3 : moderate | 4 : severe | 5 : extreme so I could do nothing |
| --- | --- | --- | --- | --- | --- |

**38. Weakness in the arm, shoulder, or hand apparently caused by the AVF**

|  | 1 : none | 2 : mild | 3 : moderate | 4 : severe | 5 : extreme so I could do nothing |
| --- | --- | --- | --- | --- | --- |

**39. Stiffness in the arm, shoulder, or hand apparently caused by the AVF**

|  | 1 : none | 2 : mild | 3 : moderate | 4 : severe | 5 : extreme so I could do nothing |
| --- | --- | --- | --- | --- | --- |

**40. Difficulty sleeping due to pain in the arm, shoulder, or hand apparently caused by the AVF**

|  | 1 : none | 2 : mild | 3 : moderate | 4 : severe | 5 : extreme so I could do nothing |
| --- | --- | --- | --- | --- | --- |

**41. Feel less capable, confident, or useful because of the AVF**

|  | 1 : none | 2 : mild | 3 : moderate | 4 : severe | 5 : extreme so I could do nothing |
| --- | --- | --- | --- | --- | --- |

**42. Concern the AVF is obstructed due to dehydration**

|  | 1 : none | 2 : mild | 3 : moderate | 4 : severe | 5 : extreme so I could do nothing |
| --- | --- | --- | --- | --- | --- |

**43. Care not to hit the arm**

|  | 1 : none | 2 : mild | 3 : moderate | 4 : severe | 5 : extreme so I could do nothing |
| --- | --- | --- | --- | --- | --- |

**44. Care not to rub the arm strongly**

|  | 1 : none | 2 : mild | 3 : moderate | 4 : severe | 5 : extreme so I could do nothing |
| --- | --- | --- | --- | --- | --- |

**45. Care to avoid insect bites on the arm**

|  | 1 : none | 2 : mild | 3 : moderate | 4 : severe | 5 : extreme so I could do nothing |
| --- | --- | --- | --- | --- | --- |

**46. Puncture site itchiness**

|  | 1 : none | 2 : mild | 3 : moderate | 4 : severe | 5 : extreme so I could do nothing |
| --- | --- | --- | --- | --- | --- |

**47. Listlessness in the shoulder after dialysis**

|  | 1 : none | 2 : mild | 3 : moderate | 4 : severe | 5 : extreme so I could do nothing |
| --- | --- | --- | --- | --- | --- |

**48. Care to protect the arm from becoming cold**

|  | 1 : none | 2 : mild | 3 : moderate | 4 : severe | 5 : extreme so I could do nothing |
| --- | --- | --- | --- | --- | --- |

**B) During dialysis**

**Please rate how much the AVF disturbed the following activities during dialysis in the past week.**

**In this part, AVF means AVF connected to the dialysis machine. If you did not have the opportunity to perform an activity in the past week, please give your best estimate on which response would be the most accurate. It does not matter which hand or arm you used to perform the activity; please answer based on your ability regardless of how well you performed the task.**

**1. Operate a mobile phone or smart phone**

|  | 1 : no difference with the AVF | 2 : mild difficulty due to the AVF | 3 : moderate difficulty due to the AVF | 4 : severe difficulty due to the AVF | 5 : not possible due to the AVF |
| --- | --- | --- | --- | --- | --- |

**2. Eat or drink**

|  | 1 : no difference with the AVF | 2 : mild difficulty due to the AVF | 3 : moderate difficulty due to the AVF | 4 : severe difficulty due to the AVF | 5 : not possible due to the AVF |
| --- | --- | --- | --- | --- | --- |

**3. Operate a TV remote controller**

|  | 1 : no difference with the AVF | 2 : mild difficulty due to the AVF | 3 : moderate difficulty due to the AVF | 4 : severe difficulty due to the AVF | 5 : not possible due to the AVF |
| --- | --- | --- | --- | --- | --- |

**4. Write**

|  | 1 : no difference with the AVF | 2 : mild difficulty due to the AVF | 3 : moderate difficulty due to the AVF | 4 : severe difficulty due to the AVF | 5 : not possible due to the AVF |
| --- | --- | --- | --- | --- | --- |

**5. Read a book**

|  | 1 : no difference with the AVF | 2 : mild difficulty due to the AVF | 3 : moderate difficulty due to the AVF | 4 : severe difficulty due to the AVF | 5 : not possible due to the AVF |
| --- | --- | --- | --- | --- | --- |

**6. Communicate with staff or other patients**

|  | 1 : no difference with the AVF | 2 : mild difficulty due to the AVF | 3 : moderate difficulty due to the AVF | 4 : severe difficulty due to the AVF | 5 : not possible due to the AVF |
| --- | --- | --- | --- | --- | --- |

**7. Remove something from your bag**

|  | 1 : no difference with the AVF | 2 : mild difficulty due to the AVF | 3 : moderate difficulty due to the AVF | 4 : severe difficulty due to the AVF | 5 : not possible due to the AVF |
| --- | --- | --- | --- | --- | --- |

**8. Take medicine**

|  | 1 : no difference with the AVF | 2 : mild difficulty due to the AVF | 3 : moderate difficulty due to the AVF | 4 : severe difficulty due to the AVF | 5 : not possible due to the AVF |
| --- | --- | --- | --- | --- | --- |

**9. Scratch an itch**

|  | 1 : no difference with the AVF | 2 : mild difficulty due to the AVF | 3 : moderate difficulty due to the AVF | 4 : severe difficulty due to the AVF | 5 : not possible due to the AVF |
| --- | --- | --- | --- | --- | --- |

**10. Sleep in an unrestricted position**

|  | 1 : no difference with the AVF | 2 : mild difficulty due to the AVF | 3 : moderate difficulty due to the AVF | 4 : severe difficulty due to the AVF | 5 : not possible due to the AVF |
| --- | --- | --- | --- | --- | --- |

The questionnaire is over. Thank you.
